# Indoor Measurement of Contact Stress Distributions for a Slick Tyre at Low Speed

**DOI:** 10.3390/s25134193

**Published:** 2025-07-05

**Authors:** Gabriel Anghelache, Raluca Moisescu

**Affiliations:** Automotive Engineering Department, National University of Science and Technology POLITEHNICA Bucharest, 060042 București, Romania

**Keywords:** tyre–road contact patch, motorsport slick tyre, stress distributions, complex transducer, inflation pressure, vertical load, camber angle, toe angle

## Abstract

**Highlights:**

**What are the main findings?**

- Complex transducer with a transversal array of sensing pins on the entire footprint width.
- Experimental programme with different values of tyre inflation pressure, vertical load, camber angle and toe angle.

**What is the implication of the main finding?**

- Tyra–road contact stress measured indoors at low speed.
- Measurements performed for motorsport slick tyres at low longitudinal speed in free-rolling conditions.

**Abstract:**

The paper presents results of experimental research on tyre–road contact stress distributions, measured indoors for a motorsport slick tyre. The triaxial contact stress distributions have been measured using the complex transducer containing a transversal array of 30 sensing pins covering the entire contact patch width. Wheel displacement in the longitudinal direction was measured using a rotary encoder. The parameters allocated for the experimental programme have included different values of tyre inflation pressure, vertical load, camber angle and toe angle. All measurements were performed at low longitudinal speed in free-rolling conditions. The influence of tyre functional parameters on the contact patch shape and size has been discussed. The stress distributions on each orthogonal direction are presented in multiple formats, such as 2D graphs in which the curves show the stresses measured by each sensing element versus contact length; surfaces with stress values plotted as vertical coordinates versus contact patch length and width; and colour maps for stress distributions and orientations of shear stress vectors. The effects of different parameter types and values on stress distributions have been emphasised and analysed. Furthermore, the magnitude and position of local extreme values for each stress distribution have been investigated with respect to the above-mentioned tyre functional parameters.

## 1. Introduction

Almost all forces controlling the dynamics of road vehicles are generated and applied in the tyre–road contact patch. These forces determine the braking, traction, free-rolling, and cornering behaviour of the vehicle, but also the stability, manoeuvrability and safety.

The forces in the tyre–road contact patch are not applied concentratedly, but they are spread on the entire contact patch area as 3D stress distributions (stress being defined as force ratio to unit area). In real rolling conditions, the stress distributions control vehicle movement. Almost all analytical/numerical models involving tyre–road contact take into account the concentrated forces in view to reduce the complexity of the simulation. In fact, each global force can be obtained from the area integral of stress distribution in the respective direction. Unfortunately, it is impossible to calculate stress distribution starting from global force.

The vertical stress in the contact patch represents pressure applied by the tyre to the road surface, and increased values of vertical stress lead to road damage. The longitudinal and lateral friction coefficients in the contact patch are obtained by dividing the correspondent force by the normal force, both in the case of global forces and in the case of forces per unit area. Simultaneous measurement of the 3D stress distributions allows calculating the friction coefficient distributions. Friction coefficients have a strong impact on force and torque generation but also on rubber slip in the contact patch, a phenomenon which leads to uneven wear of the tyre tread and road surface.

The experimental research of stress distributions in the tyre–road interface is a complex action, and it is very difficult to perform because the real contact of the tyre with the road must be maintained unaltered when the appropriate sensors are included.

There are numerous requirements for the measurement systems used for experimental research of stress distributions in the tyre–road interface: ensuring a high measurement resolution with respect to the tyre tread design, measuring the tyre–road stress distributions simultaneously in three orthogonal directions, allowing investigation of diverse tyre types (from passenger cars to trucks and airplanes), working in laboratory and in road conditions, allowing a wide range of wheel speeds over the sensing area, working in different rolling conditions (traction, braking, and cornering) on dry or wet roads, etc.

An overview of the main equipment developed worldwide up to 2010 for the measurement of tyre–road stress distributions is presented in [1]. In most cases, the sensing elements for stress distribution are placed in the road. In the last decade, only a small number of new or updated measuring systems were presented in the literature. Also, very few experimental stress distributions in the contact patch of a slick tyre have been shown.

An important modernised sensing element was presented by De Beer and Fisher [2]. This type of strain-gauged sensor was dimensionally optimised and is used for creating a linear transverse array of 21 sensing elements included in the “Stress-In-Motion (SIM)” system for measuring tyre-pavement contact stresses on a textured road surface. Each sensor measures tri-axial loads of truck or aircraft tyres. Referring to the SIM sensor, Li and Hao [3] affirmed that the contact forces are not uniformly distributed. The non-uniform distribution of vertical contact force causes a bending moment which can lead to significant errors in the tri-axial contact stress measurements. They argued that both the SIM sensing element design and the decoupling method between the measured signals should be carefully reconsidered. Xie and Yang [4] have verified their static truck tyre model using a pressure sensor array inserted in a rectangular specimen of asphalt concrete.

In recent years, the method of measuring the distribution of pressure in the contact area, using commercially available thin-layer pressure sensors, has become widespread. The results are used mainly for tyre and road model validation but also for investigating the influence of different tyre exploitation parameters on the contact pressure distribution. The pressure distribution within the tyre contact patch can be measured by introducing a pressure-sensitive film in the contact zone, as mentioned by Wallaschek and Wies [5], Lopez Arteaga and van der Steen [6], Hamlat et al. [7], Costanzi et al. [8], etc. The method is cost-efficient, simple, and reliable but can be used only for stationary tyre.

For a rotating tyre, resistive sensor technology is applied to measure the pressure distribution in the contact patch. TireScan^TM^ system [9] can investigate the pressure distribution at speeds up to 265 km/h. An application of resistive-based technology to measure the pressure distribution is shown by Leiva-Villacorta et al. in [10].

Capacitive sensing elements are able to measure pressure by inducing a change in capacitance correlated to the change in pressure. Cork [11] affirms that the sensor element is accurate, thin, flexible, and robust. Also, these sensors provide 1.15 mm resolution over the contact patch area for a total of 65,536 sensing points in the matrix. Guo et al. [12] proposed a multilayered capacitive tactile sensor sheet, which is made entirely of polymer materials, to simultaneously achieve high measurement precision and resolution.

In recent decades there have also been some achievements with sensors placed in the tyre. The physical quantities that can be measured are tyre carcass or tread deflection, tyre strain, tread acceleration, etc., resulting in a so-called “intelligent tyre”. These layouts do not offer direct but indirect measurement of the tyre–road stress distributions. Also, these distributions are not detailed and provide somewhat lower resolution. The most affected is the lateral distribution because detection of the physical quantity is generally made only in a single point located in the tyre tread transversal direction. Furthermore, instrumentation modifies locally the tyre behaviour, and difficulties arise because of power supply and signal transmission for rolling tyres. Despite these inconveniences, the instrumentation of the tyre can provide significant information related to the contact patch forces and the driving conditions, information necessary for the vehicle safety systems and also for autonomous vehicle control.

Lundberg et al. [13] presented a novel test rig design which enables detailed studies of the three force components generated in the rolling contact between a tyre tread block and a substrate. The “tyre” consists of a solid metal wheel in which a force link is embedded, consisting of an inner pre-tensioning element, a three-axial piezoelectric force transducer and an outer pre-tensioning element. The test rig enables force measurements at high rolling speeds.

Niskanen and Tuononen [14] used three accelerometers attached to the inner liner of the tyre carcass for studying contact patch deformations in wet conditions. The acceleration sensors predict the shortening of the contact length of the tyre before the critical aquaplaning speed is reached. Teerhuis et al. [15] have equipped a tyre with an accelerometer on the inner liner for measuring radial acceleration, and the experimental data was used to validate a tyre state estimator model.

Tuononen [16] presented a tyre instrumentation design for measuring tyre carcass deflections with respect to the rim, based on optical detection of position. The carcass deflections can be used to calculate tyre vertical and lateral forces, and they can be exploited in the estimation of friction. Also, Tuononen [17] proposes a method to evaluate the lateral state of a vehicle based on tyre forces which are estimated from optically measured tyre carcass deflections. Next, Tuononen introduced in [18] a laser tyre sensor which can measure the carcass deflections of a rolling tyre. Further, a laser-based sensor system is presented in [19] to measure tread block deformation.

Roveri et al. [20] described a technology for optical strain measurements in rolling tyres. The rig is based on the use of fibre Bragg grating sensors and a light spectrum analyser. The experiments are able to provide evidence of the transition along the contact patch between the grip and the slip regions.

Recently, Zhao et al. [21] proposed a tyre deformation measurement method by monitoring tyre strain with long-gauge fibre Bragg grating sensors.

Another method for determining tyre tread deformation consists of measuring strains on the inner liner of the tyre tread with special strain gauge sensors. Then, starting from tyre deformations, different types of forces acting in the tyre contact patch can be obtained, and estimation for vehicle dynamics can be performed. The research team led by Garcia-Pozuelo and Olatunbosun [22] performed extensive investigations in this field. They stated that the strain values depend on the lateral force, which can be used to predict tyre slip conditions. Also, the sensor signals allow detecting a tyre’s loss of grip and estimating the lateral friction coefficient [23]. Yunta et al. [24] found that the camber angle has a significant effect on the strain signal. Garcia-Pozuelo et al. [25] validated a proposed tyre strain model by experimental results using a strain-based intelligent tyre. Mendoza-Petit et al. [26] proposed a methodology to estimate the effective radius, the length of the contact patch, and the wheel speed at the point of contact using a tyre instrumented with strain gauges.

The literature on camber angle and toe angle influence on 3D stress distributions is very limited. Paper [27] mentions some experimental results, but many recent works are based on the modelling approach [28,29,30,31,32].

In the Tyre Research Laboratory at National University of Science and Technology POLITEHNICA Bucharest, road-embedded and laboratory test rigs were designed, produced, and used for experimental investigation of the tyre–road contact stress distributions. Between 1980 and 2000 a movable table rig was developed to investigate the shear stress distributions in the contact patch of a passenger car tyre under laboratory conditions [33]. In the 2000s, a road-embedded test rig for tri-axial shear stress distributions of truck tyres was designed, patented, produced and installed in the road. Details about technical specifications of the road-embedded test rig were presented in [1]. The experimental research was performed in real rolling conditions for free-rolling and braking truck tyres, and some results were presented in [34,35]. In the same interval, the study of stress distributions continued using finite element models of truck tyres. Some results obtained through finite element modelling, as well as their comparison with experimental results, were presented in [35]. In recent years, the test rig for measuring tri-axial tyre–road contact stress distributions was installed in the laboratory, and an experimental programme was developed for investigating the stresses in the contact patch of a slick tyre.

The first objective of this paper is to present the measurement system for contact stress distributions for a slick tyre at low speeds. The second objective is to present and discuss stress distributions measured indoors in three orthogonal directions in the contact patch at different vertical loads, inflation pressures, camber angles, and toe angles.

## 2. Tyre–Road Contact Stress Measuring System

### 2.1. Measuring System Characteristics

**Figure 1:**
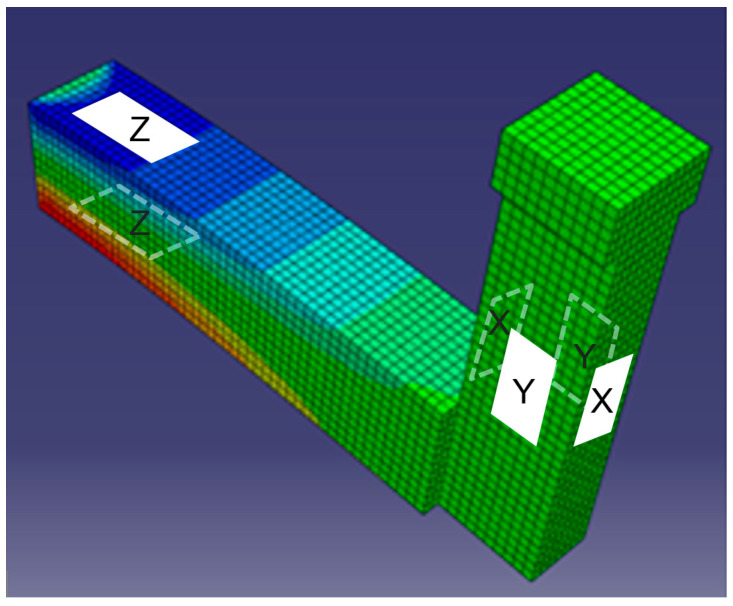
Sketch of a sensing pin.

The tyre stress measuring system presented in [1,34,35] was developed at the National University of Science and Technology POLITEHNICA Bucharest. It is capable of outdoor/indoor measurements of 3D stress distributions in real free-rolling/traction/braking conditions for different speed ranges and covering the entire contact patch width. The complex transducer is designed to provide a minimum change in contact surface, and it was initially designed for truck tyres, but it is also suitable for passenger car tyres. Figure 1 shows the sketch of a sensing pin instrumented with three strain-gauged half bridges, each measuring in the longitudinal (*X*), lateral (*Y*), and vertical (*Z*) directions. The main pin dimensions were designed through software optimisation for minimizing size and deflections under the loads but maximising sensibility. The pin body is placed in the road, and the upper area is aligned with the road surface.

In real rolling conditions, the forces acting in the tyre–road contact patch are distributed as stresses (force to unit area ratio, [Pa] or [kPa]). Each sensing pin was designed to measure force in three orthogonal directions. Knowing that the pin upper surface has a contact area of 10 mm × 10 mm, by measuring the 3D forces applied on this area, the average stresses on 100 mm^2^ can be obtained. For instance, a 50 N force applied on the upper surface of the sensing pin would be equivalent to a 500 kPa contact stress.

**Figure 2:**
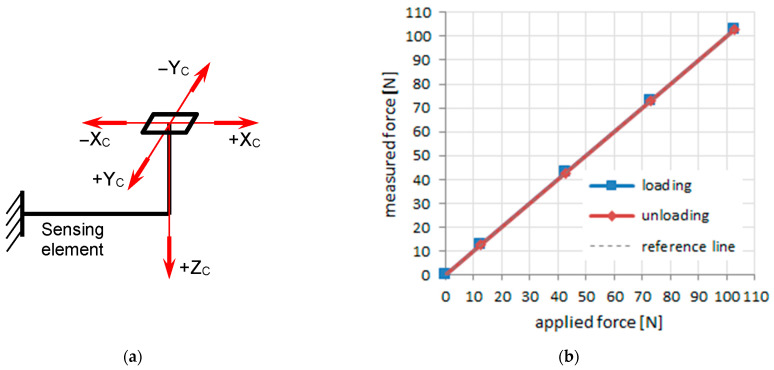
Calibration of sensing pins: (**a**) orthogonal calibration forces applied on the sensing pin; (**b**) example of linearity curves for *Z* direction.

A special rig was designed for indoor static calibration of the complex transducer. Orthogonal calibration forces, adapted for passenger car tyres, were applied on the upper surface of each sensing pin shown in Figure 2a. An example of a calibration graph, in the *Z* direction, for increasing and decreasing calibration forces, is shown in Figure 2b. The maximum values of linearity errors were up to 3% for directions *X*, *Y*, and *Z*. More details about the static calibration procedure and dynamic calibration work are presented in [1] and in [36], respectively.

**Figure 3:**
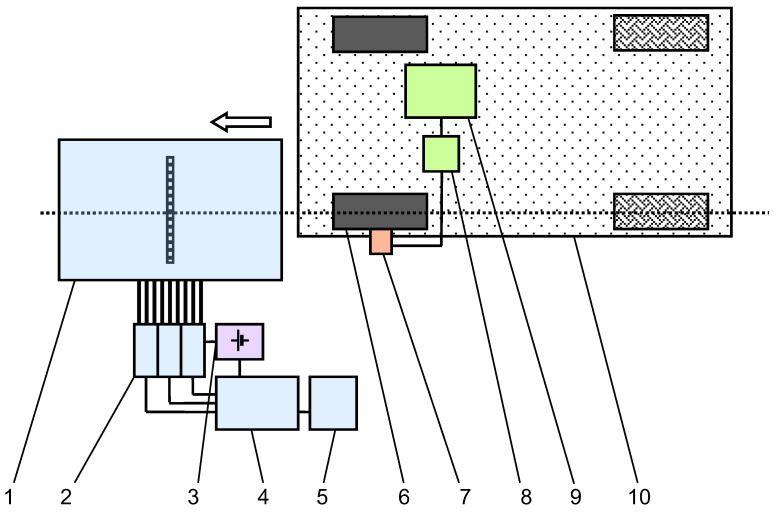
Layout of the indoor system for measuring tyre contact patch stress distributions: (1) complex transducer with array of strain gauged pins; (2) signal conditioning modules; (3) DC power supply; (4) data acquisition system with embedded computer; (5) monitor; (6) tested slick tyre; (7) wheel encoder; (8) mobile data acquisition system; (9) laptop; (10) passenger car.

The layout of the tyre stress measuring system developed at the National University of Science and Technology POLITEHNICA Bucharest for indoor testing is presented in Figure 3. For obtaining the 3D stress distributions, firstly, the 3D forces are simultaneously measured on 30 sensing pins. Knowing the unit reference pin area and sensitivity matrix [1], through special software application, the stress distributions are found. The forces on the unit area are measured using three strain-gauged half bridges on each sensing pin, with appropriate signal conditioning modules and data acquisition modules. The graphical representations of stress distributions are presented as functions of wheel displacement in the longitudinal direction. This displacement has been measured using a rotary encoder mounted on the tested tyre-wheel assembly. Papers [1,34,35] provide more details about the measuring system.

**Figure 4:**
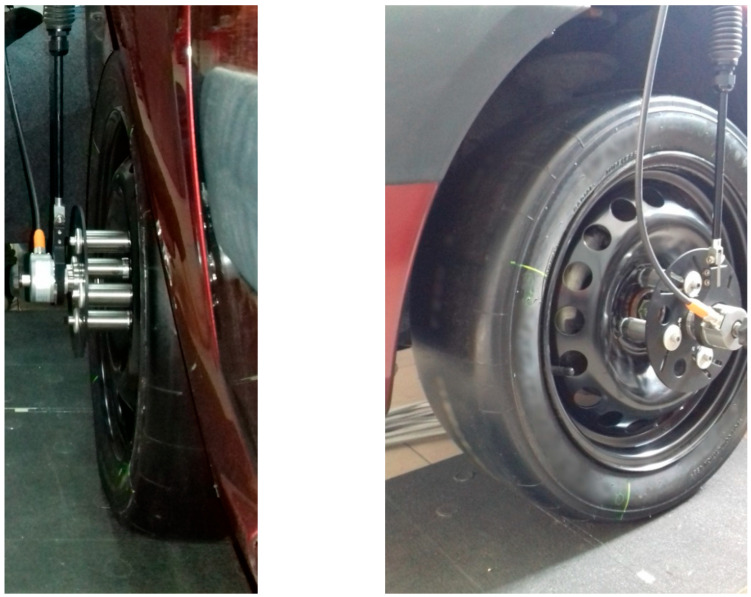
The motorsport slick tyre used for contact stress measurements.

Figure 4 contains the almost invisible transversal array of upper pin surfaces in front of the tyre contact patch. The main specifications of the measuring system are as follows: transversal array of 30 sensing pins and 90 data acquisition channels. The contact patch stress distributions have been measured with a longitudinal resolution of 2 mm resulting from the rotary encoder and a lateral resolution of 10 mm provided by the complex transducer.

### 2.2. Tyre Testing Conditions

The contact stress measurements have been performed using a 190/570R15 motorsport tyre without a tread profile (slick tyre), as shown in Figure 4, where the complex transducer and the rotary encoder mounted on the wheel can also be noticed. The reasons for testing a slick tyre refer mainly to measurements unaffected by tyre tread design but also to possible interest for motorsport specialists.

The tyre parameters allocated for the experimental programme included vertical load values in the range from 3800 N to 4400 N; inflation pressure values in the range from 160 kPa to 240 kPa; camber angles of −0.5° and +1.2°; toe angle values in the range from 0° to +6°; and a low longitudinal speed of about 1.0–1.5 km/h. The measurements have been performed in free-rolling conditions.

## 3. Stress Distributions

In this experimental research, the stress distributions represent the effect of the tyre action applied on the road. The positive and negative values indicate the orientation of each stress vector. For the longitudinal and lateral stresses, both positive and negative values appear. Instead, for the vertical stresses, the sign is always positive since they are representing pressure and they are oriented from the tyre towards the road. A positive value of longitudinal stress means that the associated vector is oriented toward the leading edge of the contact patch. A positive value of lateral stress means that the associated vector is oriented toward the inside edge of the contact patch. A zero vertical stress value appears when no contact exists between the tyre tread and the road surface. However, zero longitudinal or lateral stress values could exist even where the tyre tread is in contact with the road.

The stress distributions on each orthogonal direction are presented in several ways: as 2D graphs in which the curves represent the stresses measured by each sensing element (measuring channels mentioned in figures) versus contact length; then as surfaces with stress values plotted as vertical coordinates versus contact patch length and width; and finally, as colour maps in which the stress magnitudes and orientations are represented by colour and vector size. Observation: the vector display was not feasible for vertical stress, so instead shear stress vectors were superimposed (shear stress vectors represent the vector sum of longitudinal and lateral stresses). In this third type of representation, although the results are measured with a longitudinal resolution of 2 mm, vectors have been placed at 10 mm distance along the contact patch for improved visibility. In the first two types of representations, the orientation corresponding to positive shear stress values has been marked by arrows placed near the plots. Furthermore, the graphs offer information on the shape and dimensions of the tyre–road contact patch depending on the testing conditions.

### 3.1. Influence of Tyre Inflation Pressure

The slick tyre parameters chosen for these studies are as follows: free-rolling, inflation pressure of 160 kPa, 200 kPa, and 240 kPa, vertical load of 3800 N, −0.5° camber angle, and 0° toe angle.

**Figure 5:**
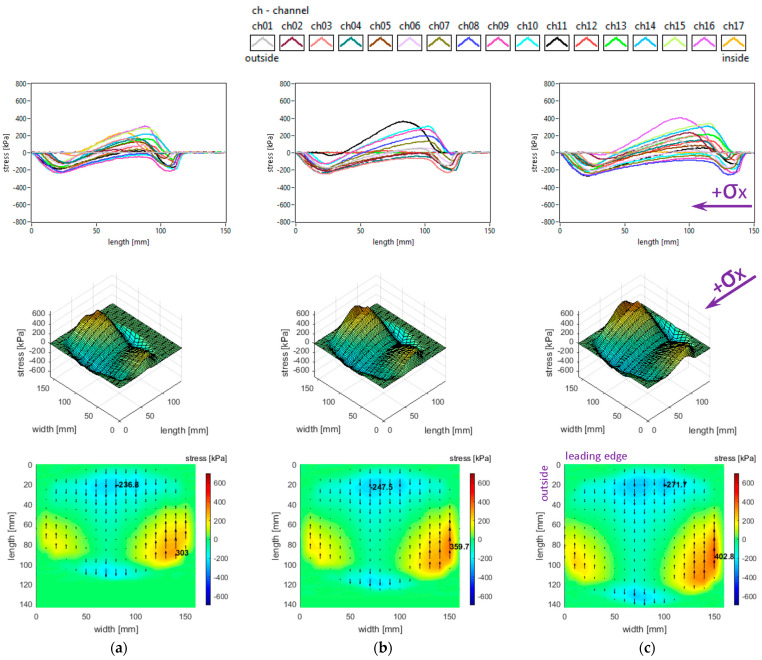
Longitudinal stress distributions at tyre load 3800 N and inflation pressure: (**a**) 240 kPa; (**b**) 200 kPa; (**c**) 160 kPa.

Generally, longitudinal stress distributions have a significant effect on tyre rolling resistance. These distributions are shown in Figure 5. It can be seen that only for small regions the longitudinal stress distributions have quasi-sinusoidal variation, while for about one third of the surface the stresses have similar orientation along the entire contact patch. The measured distributions are characterised by positive values of longitudinal stress in lateral regions, with quasi-triangular curve shapes, as well as some intensification of negative values near the trailing edge of the contact patch; overall, four distinct regions can be noticed throughout the contact patch.

Both the local extreme values of the longitudinal stress (positive and negative values in Figure 5) and the contact area increase when inflation pressure decreases. Some additional observations can be made concerning the positive values that are higher in the inner lateral region than in the outer lateral region due to wheel camber angle and also concerning the magnitude of positive longitudinal stress values that exceed the magnitude of negative values.

With respect to the measured longitudinal stress distributions, it can be asserted that these experimental results are not entirely similar to the literature. It can be noticed that they are comparable to the preliminary results published in [37] for a passenger car tyre and that the shapes of the curves show resemblances to those presented in [27,38]. The longitudinal stress distributions are similar to those presented for a slick tyre in [39] only in the areas near tyre shoulders. There are clear differences in the shapes of curves and values of longitudinal stresses with respect to [40].

**Figure 6:**
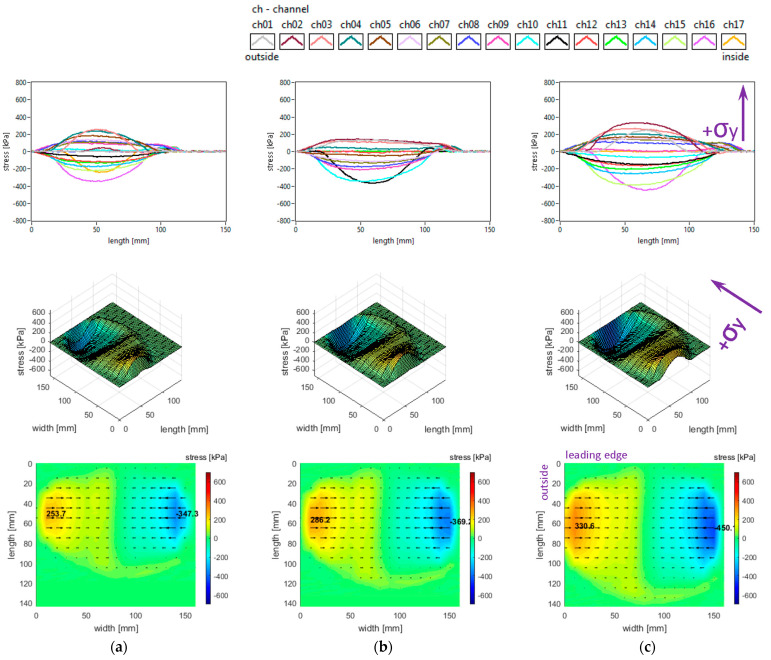
Lateral stress distributions at tyre load 3800 N and inflation pressure: (**a**) 240 kPa; (**b**) 200 kPa; (**c**) 160 kPa.

The lateral stress distributions measured in the slick tyre contact patch, for the above-mentioned parameters, are shown in Figure 6.

It can be noticed that the transversal distribution of lateral stresses has mainly a quasi-sinusoidal shape, with positive values on one side and negative values on the other side, which leads to stress orientation tending to compress the contact patch. The lateral stresses tend to exhibit a longitudinal distribution with a parabolic shape, with a similar orientation of lateral stresses along the entire contact patch.

The local extreme values of the lateral stress (positive and negative values in Figure 6) increase when inflation pressure decreases. Some additional observations can be made concerning the extreme lateral stress values that are located in the lateral regions, near the tyre shoulders, and concerning the fact that extreme lateral stress values are higher in the inner lateral region than in the outer lateral region due to wheel camber angle.

From the point of view of the measured lateral stress distributions, it can be asserted that these experimental results are quite similar to the literature. It can be noticed that they are comparable to the preliminary results for a passenger car tyre published in [37] and close to the results presented in [27,40]. Furthermore, the lateral stress distributions are similar to those presented for a slick tyre in [39].

**Figure 7:**
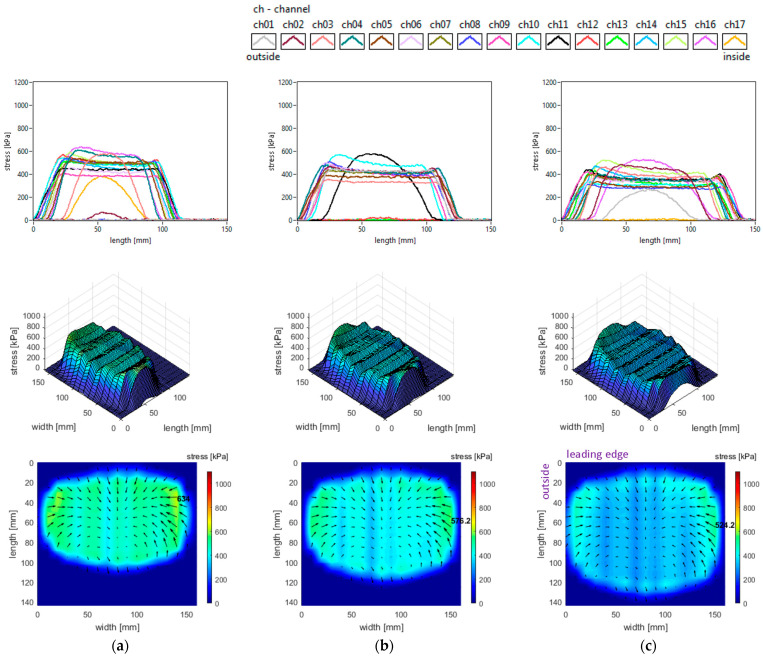
Vertical stress distributions at tyre load 3800 N and inflation pressure: (**a**) 240 kPa; (**b**) 200 kPa; (**c**) 160 kPa.

The vertical stress distributions measured in the slick tyre contact patch, for the above-mentioned parameters, are shown in Figure 7.

These graphs allow better investigation of the shape and dimensions of the contact patch. It can be seen that the contact patch has a larger width than length. Also, the contact patch length is augmented by decreasing tyre inflation pressure. However, the contact patch width is very little increased by reducing tyre inflation pressure. This can be explained by the radial construction of the tested tyre.

It can be seen that the vertical stress curves have a typical shape (trapezoidal or parabolic) with a slightly descending area between two local peaks. The maximum values are much higher than the tyre inflation pressure, and they increase when inflation pressure increases.

Some additional observations can be made concerning the high values in the lateral regions, near the tyre shoulders, and concerning the fact that maximum values are located in the inner lateral region due to wheel camber angle.

Also, from the point of view of the measured vertical stress distributions, it can be asserted that these experimental results are quite similar to the literature. It can be noticed that they are comparable to the preliminary results for a passenger car tyre published in [37]. The distributions in the tyre–road contact patch are similar to those presented in [5] for glass surfaces and in [9]. Also, comparable results are shown in [40], and somewhat close results are shown in [27]. The vertical stress distributions are similar to those presented for a slick tyre in [39].

It can also be noticed that shear stress results are not very similar to the literature taking into consideration the “fountain” shape of vector distributions representing the action of the tyre upon the road. The shear stress distributions are similar to those presented for a slick tyre in [39] only in the inside half and trailing area of the contact patch.

### 3.2. Influence of Tyre Load

**Figure 8:**
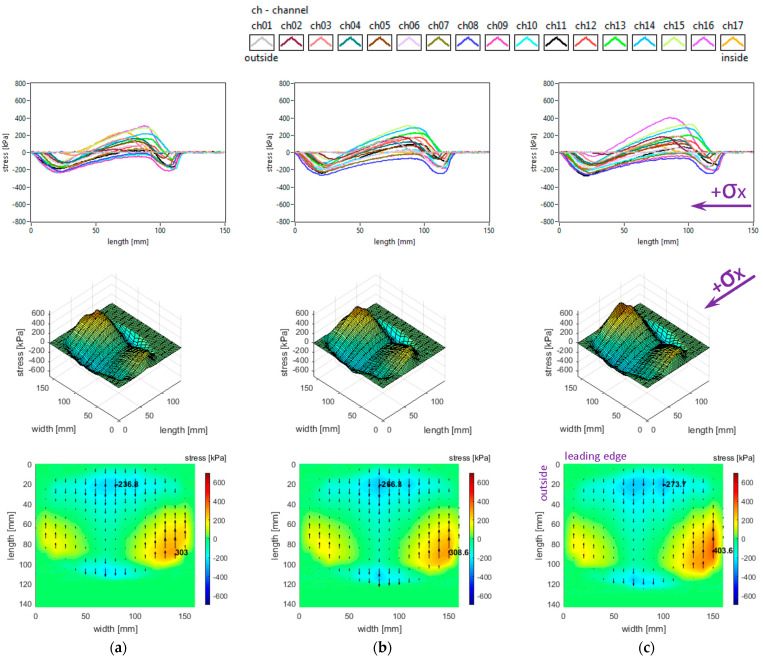
Longitudinal stress distributions at inflation pressure 240 kPa and tyre load: (**a**) 3800 N; (**b**) 4100 N; (**c**) 4400 N.

The slick tyre parameters chosen for these studies are as follows: free-rolling, inflation pressure 240 kPa, vertical load 3800 N, 4100 N, and 4400 N, −0.5° camber angle, and 0° toe angle. The longitudinal stress distributions are shown in Figure 8.

It can be seen that longitudinal stress values tend to increase slightly when the vertical load increases (including the absolute values of local extreme stresses). Other graphical aspects of the longitudinal stress distributions are quite similar to those mentioned in Section 3.1. Some curves and values are comparable to those presented in [41].

**Figure 9:**
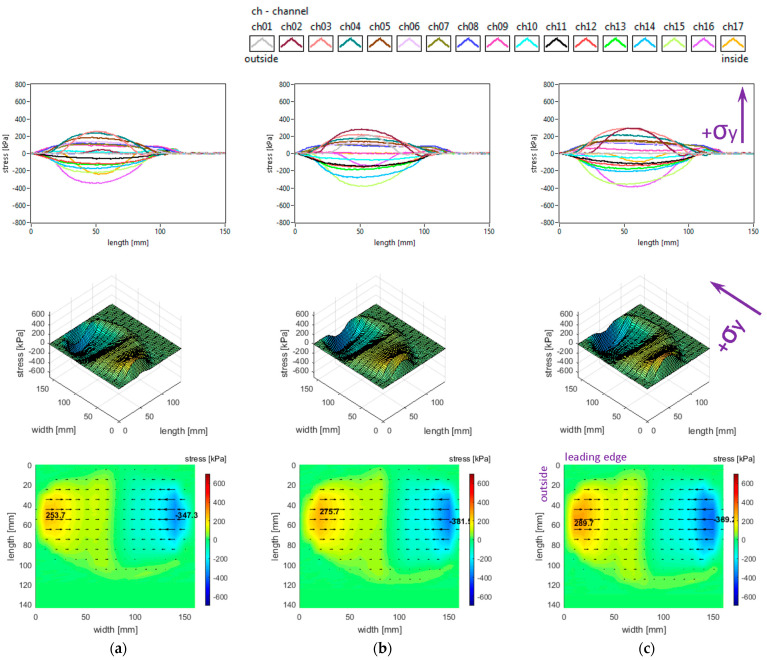
Lateral stress distributions at inflation pressure 240 kPa and tyre load: (**a**) 3800 N; (**b**) 4100 N; (**c**) 4400 N.

The lateral stress distributions measured in the slick tyre contact patch for the above-mentioned parameters, are shown in Figure 9. It can be remarked that lateral stress values tend to increase when the vertical load increases (including the absolute values of local extreme stresses). Other graphical aspects of the lateral stress distributions are quite similar to those mentioned in Section 3.1. Some similarity to the experimental values shown in [41] can be found, but the mentioned reference presents only results in the central longitudinal area of the contact patch.

**Figure 10:**
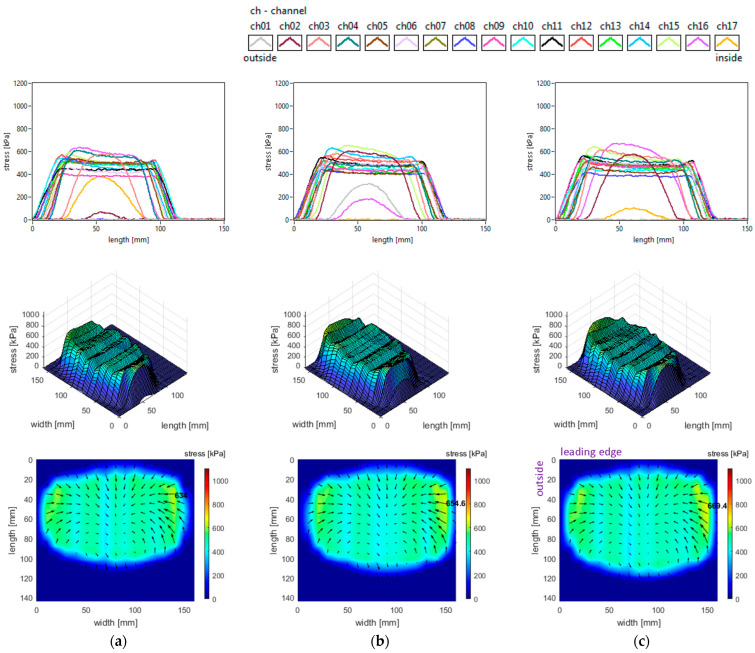
Vertical stress distributions at inflation pressure 240 kPa and tyre load: (**a**) 3800 N; (**b**) 4100 N; (**c**) 4400 N.

The vertical stress distributions measured in the slick tyre contact patch, for the above-mentioned parameters, are shown in Figure 10. The contact patch length is considerably smaller than its width, and it slightly increases when tyre load increases. The contact patch shape remains almost unchanged when tyre load varies in the mentioned interval. The increase in tyre vertical load leads to a small increase in vertical stress magnitudes (including maximum values) and small changes in the vertical stress distributions. Other graphical aspects of the vertical stress distributions are quite similar to those described in Section 3.1. Some curves and values show resemblances to those presented in [41], and also the influence of tyre load is comparable. Quite similar vertical stress distributions obtained experimentally are shown in [42].

It is noteworthy that the vector shear stress distributions (representing the action of the tyre upon the road) have “fountain” shapes which differ from the graphs mentioned in the literature [27].

### 3.3. Local Extreme Stress Values

The slick tyre parameters chosen for these comparisons are as follows: free-rolling, inflation pressure of 160 kPa, 200 kPa, and 240 kPa; vertical load of 3800 N, 4100 N, and 4400 N; a −0.5° camber angle; and a 0° toe angle. The dependencies of extreme stress values vs. inflation pressure and vertical load are analysed, taking into consideration additional data from the testing programme.

**Figure 11:**
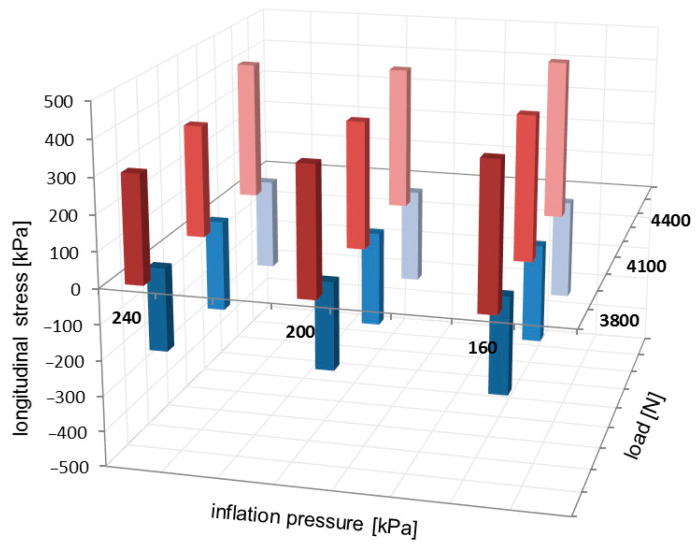
Comparison of local extreme values for longitudinal stress distributions.

Examples of the local extreme value locations on the longitudinal stress distributions have been highlighted in Figure 5 and Figure 8. The negative extreme values are placed towards the leading edge of the contact patch. Due to the negative camber angle, the positive extreme values of longitudinal stresses are located towards the inner region of the contact patch. The comparison of local extreme values for the longitudinal stress distributions is presented in Figure 11.

For longitudinal stress distributions, local extreme values in Figure 11 slightly increase when the inflation pressure decreases and when the vertical load increases. Also, the positive extreme values considerably exceed the negative magnitudes.

**Figure 12:**
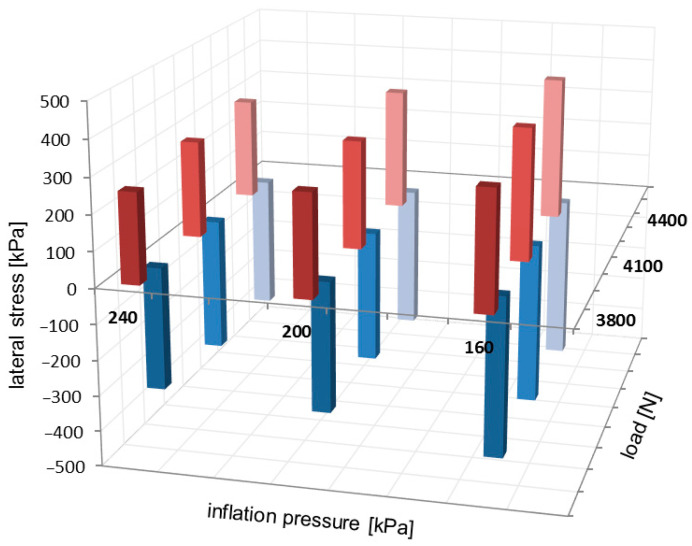
Comparison of local extreme values for lateral stress distributions.

Examples of the local extreme value locations on the lateral stress distributions have been highlighted in Figure 6 and Figure 9. The positive extreme values of lateral stresses are placed towards the outer region of the contact patch, and the negative ones are located towards the inner region of the contact patch. Due to the negative camber angle, the highest values of the extreme lateral stress are located toward the inner region of the contact patch. The comparison of local extreme values for the lateral stress distributions is presented in Figure 12.

For lateral stress distributions, local extreme values in Figure 12 slightly increase when the inflation pressure decreases and when the vertical load increases. Also, the negative extreme values considerably exceed the positive magnitudes.

**Figure 13:**
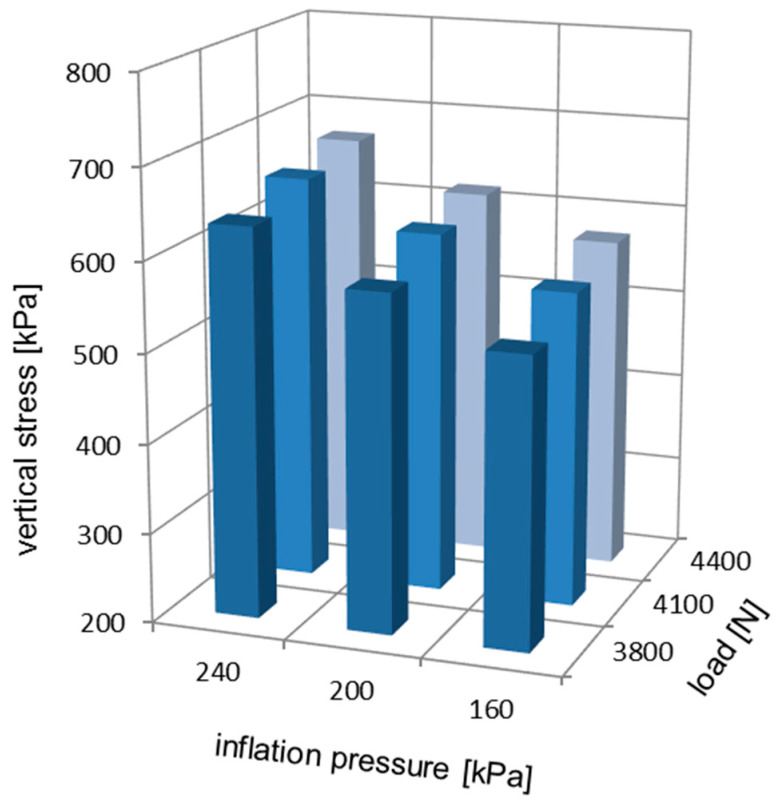
Comparison of local extreme values for vertical stress distributions.

Examples of the local extreme value locations on the vertical stress distributions have been highlighted in Figure 7 and Figure 10. Due to the negative camber angle, the extreme values of vertical stresses are located towards the inner region of the contact patch. The comparison of the local extreme values for the vertical stress distributions is presented in Figure 13.

The vertical stress magnitudes in Figure 13 augment when the inflation pressure and the vertical load increase.

### 3.4. Influence of Camber Angle

**Figure 14:**
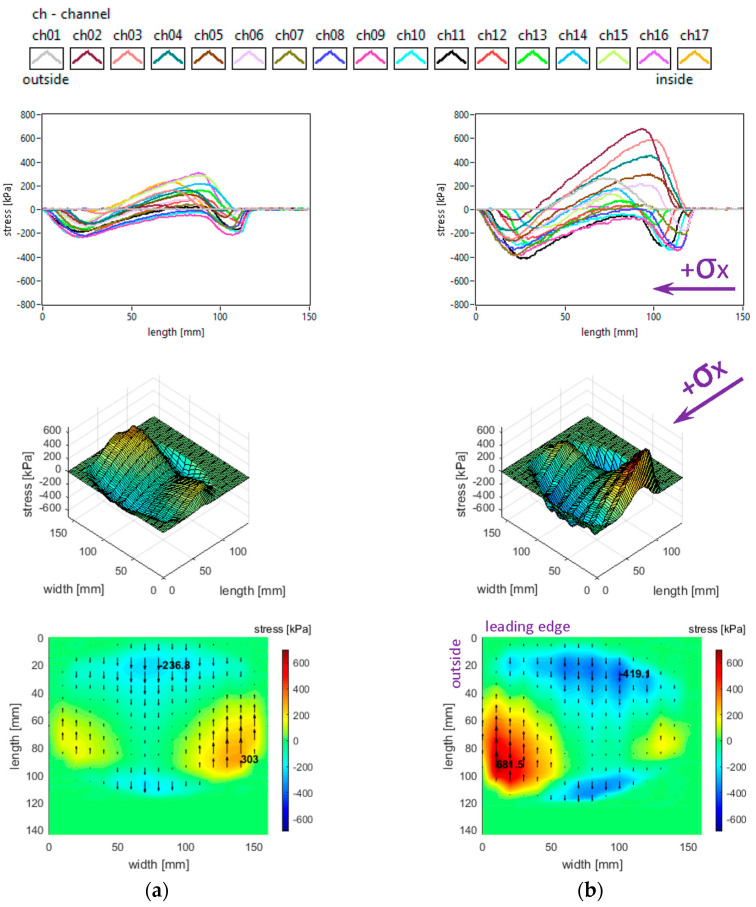
Longitudinal stress distributions at camber angles of: (**a**) −0.5°; (**b**) +1.2°.

The slick tyre parameters chosen for these studies are as follows: free-rolling, inflation pressure 240 kPa, vertical load 3800 N, −0.5° and +1.2° camber angles, and 0° toe angle. The longitudinal stress distributions are shown in Figure 14. The change in camber angle from −0.5° to +1.2° induces important modifications in the longitudinal stress distributions. A notable non-symmetry affects the longitudinal stress distribution. Although the four regions in Figure 5 can be recognised also in Figure 14, these areas are very much altered, with an increase in the distinct areas and of the maximum values, especially on the outer lateral, leading edge and trailing edge of the contact patch.

The maximum lateral value moves from the inner region to the outer region. All local extreme values are almost doubled in the leading edge and in the outer lateral area.

**Figure 15:**
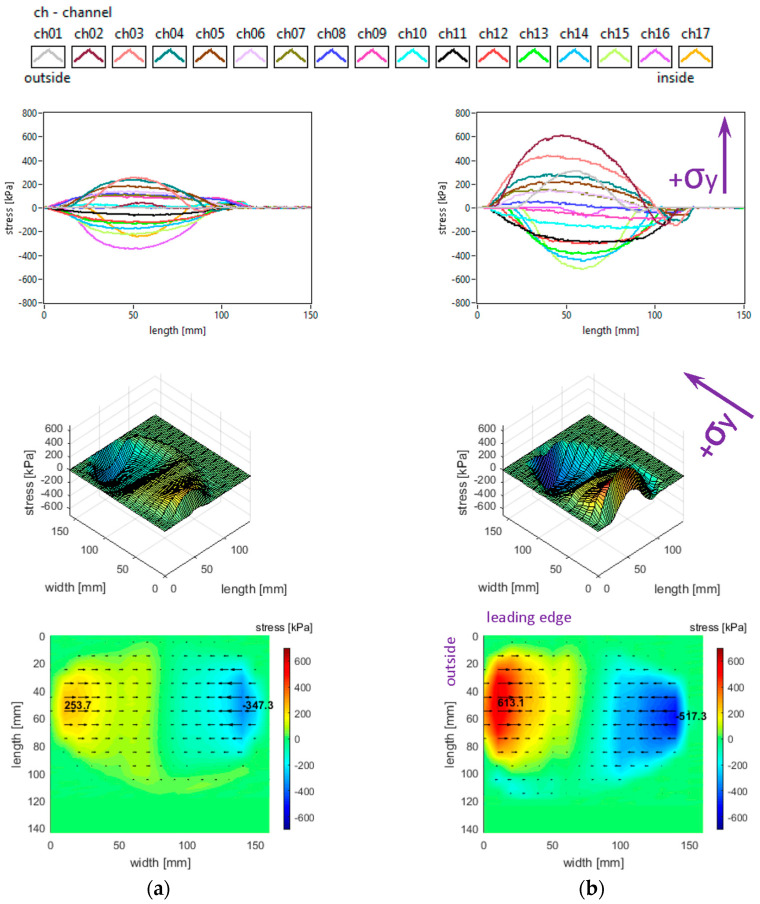
Lateral stress distributions at camber angles of: (**a**) −0.5°; (**b**) +1.2°.

The lateral stress distributions measured in the slick tyre contact patch, for the above-mentioned parameters, are shown in Figure 15. Compared to the data in Figure 6, the increase in camber angle does not change the stress curve’s shape, but it introduces a non-symmetry in stress distributions, showing a strong increase in lateral stress values both in the outer and inner regions of the contact patch. Results alike are shown in [27]. The stress extreme value migrates from the inner to the outer region of the contact patch, and it is more than twice greater in magnitude.

**Figure 16:**
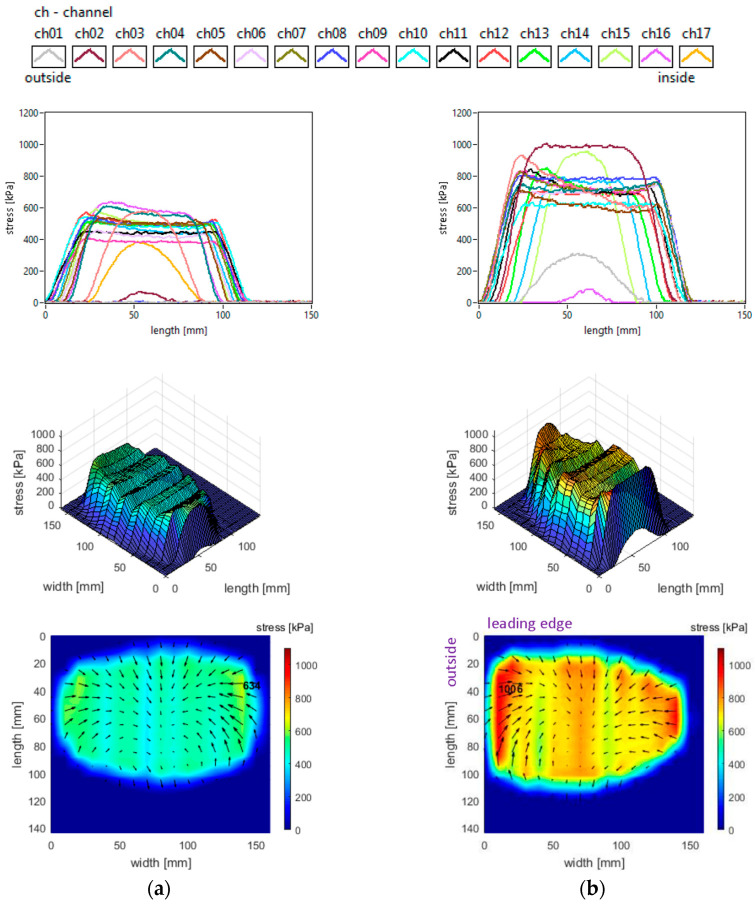
Vertical stress distributions at camber angles of: (**a**) −0.5°; (**b**) +1.2°.

The vertical stress distributions measured in the slick tyre contact patch, for the above-mentioned parameters, are shown in Figure 16. Compared to the data in Figure 7, the increase in camber angle modifies the shape and increases the length of the contact patch, especially in the central and outer regions. Paper [27] specifies similar results, and paper [28] mentions such behaviour for a modelled tyre.

Furthermore, the comparison shows that the vertical stress curve shape remains the same, but the magnitudes strongly increase. On almost the entire contact patch area, the vertical stress values exceed the maximum value obtained for the small camber angle. The maximum value is about 60% greater and moves from the inner region to the outer zone of the contact patch. These results are partially confirmed by those in [30] for a brush tyre model and in [31], but for a modelled aircraft tyre. The maximum value is more than four times higher than the tyre inflation pressure.

It can also be noticed that the “fountain” shape of shear stress vector distributions is clearly non-symmetrical, appearing especially on the outer half part of the contact patch.

The results regarding contact patch shape alteration and shear stress distributions due to camber angle are partially comparable to those obtained by modelling for a racing tyre published in [43].

### 3.5. Influence of Toe Angle

**Figure 17:**
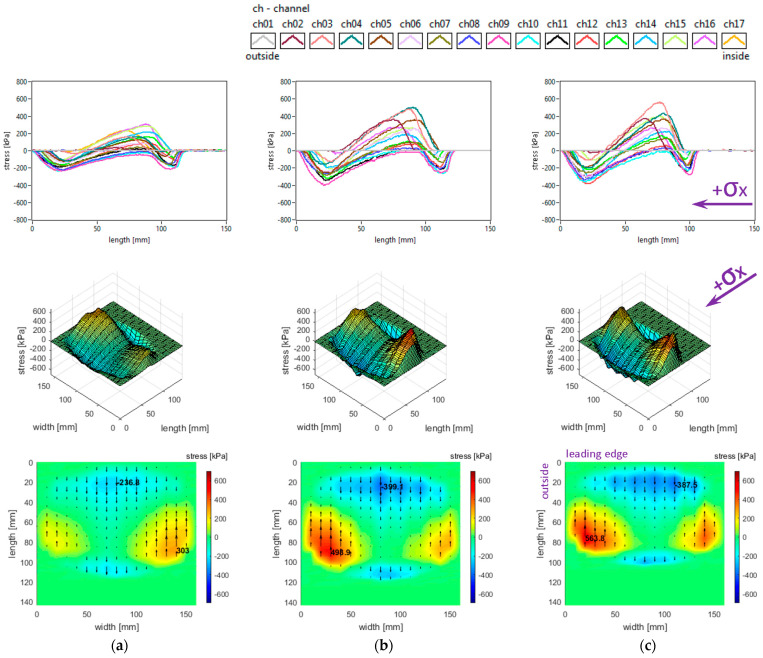
Longitudinal stress distributions at toe angles of: (**a**) 0°; (**b**) +3°; (**c**) +6°.

The slick tyre parameters chosen for these studies are as follows: free-rolling, inflation pressure 240 kPa, vertical load 3800 N, and toe angles of 0°, +3°, and +6° (toe-in). The longitudinal stress distributions are shown in Figure 17. The increase in toe angle does not change the shape of the longitudinal stress curves but increases the stress magnitudes and introduces some non-symmetry for these distributions. Although the distinctive four regions for the longitudinal stress distributions still exist, the increase in toe angle modifies the area and the local extreme values for each region. Also, the maximum value location on the inner region (for 0° toe angle) migrates to the outer region, and the value considerably increases.

**Figure 18:**
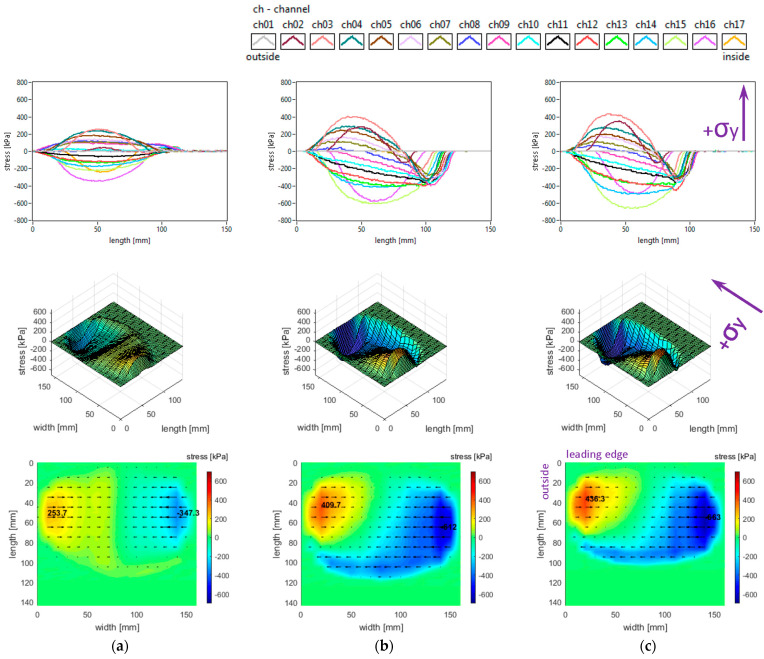
Lateral stress distributions at toe angles of: (**a**) 0°; (**b**) +3°; (**c**) +6°.

The lateral stress distributions measured in the slick tyre contact patch, for the above-mentioned parameters, are shown in Figure 18. Increasing the toe angle drastically modifies the lateral stress shapes, distributions, and values. Comparing with longitudinal and vertical stress distributions, it can be observed that the lateral ones have the largest alterations of shape and value. On the outer half of the contact patch, many longitudinal stress distribution curves change from parabolic shape to quasi-sinusoidal representations. As toe angle increases, the vector graphs of lateral stress distributions show impressive change in shapes with the “negative” areas enlarging. Consequently, it is obvious that a steering torque is generated in the contact patch. The stress local extreme values almost doubled when the toe angle was modified from 0° to 3°, but the rate of change for the maximum values was smaller for the toe angle from 3° to 6°.

**Figure 19:**
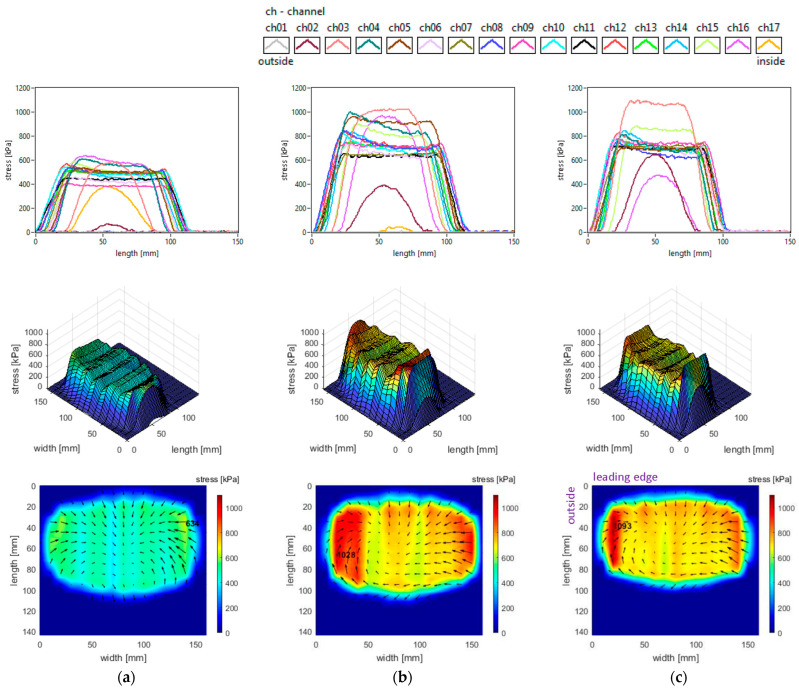
Vertical stress distributions at toe angles of: (**a**) 0°; (**b**) +3°; (**c**) +6°.

The vertical stress distributions measured in the slick tyre contact patch, for the above-mentioned parameters, are shown in Figure 19. The increase in toe angle induces small changes in the contact patch shape and a little decrease in its length. The vertical stress curve’s shape remains almost the same, but the magnitudes strongly increase. Toe angle influence is quite unavailable in the literature, but in [32] the authors mention some vertical stress distributions for slip angle simulation. The vertical stress values on most of the contact patch area exceed the maximum value obtained for the 0° toe angle. For a toe angle of 6°, the maximum value is about 72% greater and moves from the inner region to the outer zone of the contact patch. The vertical stress distributions have strong nonlinear modifications, the main alteration being observed for toe angles of 3° and 6°. Comparing the distributions for toe angles of 3° and 6°, it can be seen that larger areas with high vertical stress magnitudes appear in the case of the 3° toe angle. In both cases, the maximum values appear on the lateral regions of the contact patch, especially on the outer zone. The maximum values are more than four times higher than the tyre inflation pressure.

It can also be noticed that the shape of shear stress vector distributions is modified from “fountain” type (for toe angle 0°) to “spiral” shapes for greater toe angles, mainly due to the lateral stress distributions.

## 4. Conclusions

The literature on contact stress distributions for slick tyres is very limited, and the results published in this paper differ in some respects from those previously reported.

The paper presents and discusses stress distributions measured on three orthogonal directions in the tyre–road contact patch at different vertical loads, inflation pressures, camber angles and toe angles. The special measuring system was installed indoors, and the investigations were performed for a motorsport slick tyre in free-rolling conditions at low speeds. The contact patch stress distributions have been measured with good precision and resolution.

The contact patch shape remains almost unchanged when inflation pressure, tyre load and toe angle vary. In contrast, the increase in camber angle significantly modifies the contact patch shape. The contact patch width is very little increased by diminishing tyre inflation pressure and increasing tyre load. Its length is augmented by decreasing tyre inflation pressure, increasing tyre load, increasing camber angle (especially in the central and outer regions), and decreasing toe angle (which has little effect).

Generally, the maximum stress values in three orthogonal directions are much higher than the tyre inflation pressure.

Four distinct regions for longitudinal stress distributions can be noticed throughout the contact patch.

The absolute values of longitudinal and lateral extreme stresses increase when inflation pressure decreases and when vertical load, camber angle and toe angle increase. Also, increasing the toe angle drastically modifies the lateral stress shapes, distributions, and values.

The peaks of vertical stresses increase when inflation pressure, vertical load, camber angle, and toe angle increase.

The vector shear stress distributions have “fountain” shapes when inflation pressure and vertical load vary. When modifying camber angles, the “fountain” shape of shear stress vector distributions becomes highly non-symmetrical. By increasing the toe angle, the shape of shear stress vector distributions is modified from “fountain” type to “spiral” shapes.

Knowing these aspects, the stress distributions obtained through experimental research have an important role in tyre design, numerical tyre model validation, and optimisation of tyre parameters and wheel geometry. Recent evolutions of intelligent tyres allow incipient stages of stress distribution measurements, using sensing elements integrated into the tyre structure. This information captured in real time could contribute to developing control algorithms in autonomous vehicles and improving motorsport performances.

## Data Availability

Data are contained within the article.
